# Systems Thinking in Practice: Participatory Modeling as a Foundation for Integrated Approaches to Health

**DOI:** 10.3389/fvets.2018.00303

**Published:** 2018-12-17

**Authors:** Raphaël Duboz, Pierre Echaubard, Panomsak Promburom, Margaret Kilvington, Helen Ross, Will Allen, John Ward, Guillaume Deffuant, Michel de Garine-Wichatitsky, Aurélie Binot

**Affiliations:** ^1^ASTRE, CIRAD, INRA, Univ Montpellier, Montpellier, France; ^2^Institut Pasteur du Cambodge, Phnom Penh, Cambodia; ^3^Global Health Asia Institute, Bangkok, Thailand; ^4^Center for Agricultural Resource System Research (CARSR), Chiang Mai University, Chiang Mai, Thailand; ^5^ISREF-Independent Social Research, Evaluation & Facilitation, Christchurch, New Zealand; ^6^School of Agriculture and Food Sciences, The University of Queensland, Brisbane, QLD, Australia; ^7^Learning for Sustainability, Christchurch, New Zealand; ^8^Mekong Region Futures Institute, Bangkok, Thailand; ^9^National Research Institute of Science and Technology for Environment and Agriculture, Antony, France; ^10^Faculty of Veterinary Medicine, Kasetsart University, Bangkok, Thailand

**Keywords:** One Health, EcoHealth, Planetary Health, systems theory, systems thinking, participatory modeling, resilience, sustainable development goals

## Abstract

One Health (OH), EcoHealth (EH), and Planetary Health (PH) share an interest in transdisciplinary efforts that bring together scientists, citizens, government and private sectors to implement contextualized actions that promote adaptive health management across human, animal and ecosystem interfaces. A key operational element underlying these Integrated Approaches to Health (IAH) is use of Systems Thinking as a set of tools for integration. In this paper we discuss the origins and epistemology of systems thinking and argue that participatory modeling, informed by both systems theory and expertise in facilitating engagement and social learning, can help ground IAH theoretically and support its development. Participatory modeling is iterative and adaptive, which is necessary to deal with complexity in practice. Participatory modeling (PM) methods actively involve affected interests and stakeholders to ground the field of inquiry in a specific social-ecological context. Furthermore, PM processes act to reconcile the diverse understandings of the empirical world that stem from divergent discipline and community viewpoints. In this perspective article, we argue that PM can support systems thinking in practice and is essential for IAH implementation. Accordingly we invite PH, OH, and EH practitioners to systematically incorporate specialists in systems science and social engagement and facilitation. This will enable the appropriate contextualization of research practice and interventions, and ensure a balanced representation of the roles and relationships of medical, biological, mathematical, and social disciplines. For completeness, funding schemes supporting IAH need to follow the same iterative, adaptive, and participative processes to accompany IAH projects throughout their implementation.

## Introduction

The emergence of One Health (OH) and EcoHealth (EH) approaches over the past decade, recently complemented by the Planetary Health (PH) movement (1), illustrates the consolidation of a consensus in the veterinary and public-health domains that there is a need for integrated, interdisciplinary and inter-sectoral approaches to better understand health issues, and to improve the sustainability and relevance of interventions targeting individual and population health in various social, cultural and environmental contexts. The recent publishing of the One Health theme issue in the Philosophical Transactions of the Royal Society B (2) and the present issue embody this consensus and highlight the need for a clarification of the principles underlying these integrated approaches to health (IAH) (3, 4), the key methods and tools they rely on to collect data, monitor processes and outcomes, and their effectiveness and relevance for coping with veterinary and public health issues.

The Ecosystem approach, underpinning Ecohealth (and in principle One Health) (Wilcox et al., under review in this Research Topic), has been developed over the last two decades (4–6) to address pressing social-ecological challenges. However, it remains sparsely used when it comes to public health related interventions (Wilcox et al., under review in this Research Topic). Building on the conceptual foundation of the ecosystem approach (6), we propose that IAH initiatives, whatever their dominant epistemological orientation (e.g., One Health and veterinary sciences, Ecohealth and disease ecology, etc.), should routinely take a comprehensive view of the complex interactions between human and natural systems at multiple scales and levels (7). This would require taking a participatory approach to more widely involve key stakeholders in:

1. definition and description of relevant social-ecological systems in terms of scale, extent, structure and functioning;
2. assessment of their state in terms of health, as defined by what is acceptable to society;
3. assessment of threats;
4. maintenance, mitigation, and rehabilitation, and;
5. using adaptive management strategies to ensure longer term systems resilience (8).

We also argue for more attention within these participatory approaches to the use of modeling techniques which encourage those involved to clarify what key variables affect the underlying system(s), the factors that will shape responses, uncertainties involved, and the likely outcomes of particular strategies (9).

Such integrated approaches (to health and other areas) recognize that animals, humans and the environment are interdependent, and the complex interactions between these components not only challenges simplistic views of ecosystem functioning (10), but should change the way we manage them, moving from the imposition of technocratic solutions targeted at subsets of the system; to working directly with stakeholders in the decision process, and acknowledging local social-ecological knowledge in practice (6, 11). This is, in effect, social learning as a participatory process of social change in which people learn from each other in ways that can benefit wider social-ecological systems (12), and in which modeling serves as a key tool, both for this learning to happen and for supporting decision-making processes (13, 14).

Zinsstag et al. (3) argue that individual and population health can be seen as emerging properties of a system's interacting social and ecological factors. It follows that any IAH attempting to sustainably improve public-health and population well-being needs to help key stakeholders understand and adaptively manage social-ecological dynamics. We note two major impediments to the success of IAH projects in this regard. Firstly, there is insufficient representation and integration of social sciences and disciplines related to ecological thinking and environmental management (15–18). Secondly, more participatory and integrated modeling is needed to help systematically capture and integrate our understanding of how changes in management, climate, demographics, and other factors affect selected indicators of system health (19). The fact that these fields are underrepresented in IAH reflects the human and veterinary health sector foci on disease when addressing health issues. Disease management is not the same as promoting health, which requires a more salutogenic orientation (20, 21) grounded in resilience theory and practice, medical sociology and anthropology and participatory action-research. This orientation more closely fits what arguably should be One Health's primary concern to maintain the integrity of the human-animal-environment complexes in face of stressors. This orientation is also better aligned with the United Nations Sustainable Development Goals, which involve a more diverse range of expertise as well as methodologies for their integration (Wilcox et al., under review in this Research Topic).

In this perspective article, we highlight the importance of underpinning IAH approaches with three key practice areas: (1) systems thinking—to help a wider range of stakeholders develop a shared agreement around problem structuring; (2) modeling—to help people understand trends and see processes unfold that are either too big or too little to appreciate with the naked eye; and (3) facilitation—to support the constructive participation of key stakeholders in the wider decision-making process. We also reinforce the importance of linking across these three practice areas. We begin by discussing the converging attributes and underlying characteristics of OH, EH, and PH (all designated by “IAH”) [see also Lerner and Berg (22) for a complementary discussion] and briefly clarify system thinking origins and epistemology. Finally, we highlight a rationale for the design of more integrated and adaptive methodologies for effective implementation dealing with complexity in practice.

## The Underlying Characteristics of IAH

### Complexity

IAH recognizes that complexity is an inherent characteristic of natural systems (10, 11). Since the mid 1980s, starting with the pioneers of the Santa Fe Institute in the United States, complex systems became a field of investigation. Definitions of systems properties that are commonly associated with complexity, such as chaos, scales, emergence, bifurcation, auto-organization, adaptation, and resilience can be found in the complex systems literature. Here, we focus on some features of this field that have direct implications for IAH.

While complexity and how to address it is still debated, the synthesis by Deffuant et al. (23) offers a good overview of the origins, uses and operational implications of complexity–based practices and offers a roadmap for more integrative implementation (Table 1). The authors distinguish three main epistemological views pointing to the importance of clearly understanding and explicitly acknowledging which view(s) are being considered, and perhaps integrated, when dealing with complexity on a project-basis. These viewpoints influence the kinds of questions we raise and the methods we develop to address them (Table 1).

**Table 1:** Three views of complexity and their implications for IAH implementation—Elaborated from (23).

**View**	**Main origins**	**Main characteristics**	**Consequences**
1	• Mathematics • Physics • Computer sciences	• Sensitivity to initial conditions. • A complete/full description of system outcomes is not possible (while the rules may be simple). • For most systems, their global behavior cannot be directly inferred from the rules governing components and their interactions (holism).	• A small perturbation/event can have dramatic consequences. • Impossibility of predicting with certainty (uncertainty and errors in the predictions). • Existence of emergent properties.
2	• Data mining • Computer sciences	• Acknowledges vision 1. • Modeling and simulation to integrate heterogeneous expertise and massive data sources is the main strategy to build new knowledge. • The prediction of future states is an approximation and is confined within a certain time and space horizon.	• Decision making cannot be perfect. • We can narrow down the set of possibilities.
3	• Sociology • Cognitive sciences • Biology	• The subjectivity of individuals and societies is a difficult problem in modeling. • Ecosystems (including societies) self-regulate, adapt, evolve. • Heterogeneities in perceptions, values, regulations, and social structures.	• Importance of resilience. • Participation is required. Adaptive management is required.

The three views of complexity have different implications related to how the principles and methodological attributes are implemented in the context of IAH. View 1 acknowledges that the predictive power of models^1^ is often intrinsically limited because of the sensitivity to initial conditions, that uncertainty and surprise must be taken into account, and that emerging properties should be identified—and so far as possible understood. From view 2, it is understood that heterogeneous available knowledge and data can be integrated into simulation models used to explore scenario-based projections. Complementarily, view 3 recommends the use of participatory processes to build iterative and adaptive management strategies in which resilience thinking and the active engagement of stakeholders to insure relevant representation of worldviews and perceptions, attributes that are inherently subjective, are essential ingredients.

### Participation and Transdisciplinarity

When implementing IAH, the ultimate goal is to improve health of humans, animals and ecosystems conjointly. This statement is highly subjective, and the third vision of complexity tells us that to achieve such a goal, participation (active involvement and engagement of affected interests and stakeholders in framing the problem and the discovery of adaptive responses to meet IAH objectives) is key. Attention should be paid to the participation label. It hides a substantial diversity of inclusion criteria and practices (24, 25). In participatory research, the diversity in implementing and designing participatory processes relies on the necessity to adapt to local contexts and the classical questions regarding who to involve, how and where, and for whose benefit (26, 27). The point here is to acknowledge that representatives of civil society, and decision makers at all relevant levels, must be involved in IAH, including in research design and implementation. Accordingly, the mobilized knowledge is not only scientific and expert, but also practical and local (28, 29). Consequently, power relations (who knows, decides and acts) are decentralized, shared between heterogeneous actors, and made dynamic throughout the participatory process (24, 30). Winter [(31), p7] contends that “*policy* [substituted here as IAH] *brings to statement what is judged to be possible, desirable and meaningful… and is the nexus of facts, value and ultimate meaning in which scientific, ethical and theological-philosophical reflections meet*.” Embracing the complexity of a situation with its heterogeneous set of actors, accounting for the diversity of their perspectives, perceptions and values, creating knowledge oriented toward solutions and transferable to both scientific and societal practice, is fundamental to transdisciplinary research (32–36). IAH practice should also encourage researchers to actively include a process of reflection about the transdisciplinary process itself (25). Allen et al. (37) and Seidl (38) contend that the majority of researchers in the “hard” sciences are unfamiliar with such a reflexive approach and may consider it a challenge to existing power relationships, but it is essential to the progress of the field.

Transdisciplinary research bridges science and practice (39, 40) and fosters the emergence of social learning and collective intelligence via participation. Boulding (41) argues that effective applications of general systems theory (and by extension transdisciplinary research and participatory modeling) catalyzes and coheres a “Republic of Learning” comprised of all affected interests and disciplines.

The extent to which the historical, cultural, environmental, sociological, and economical contexts are integrated depends on the definition of the problem and the formation of an interdisciplinary, cross-sectoral team that will define it. Lerner and Berg (22) argue the definition of boundaries when adopting an integrated approach is an important step. The same can be said regarding the choices in the levels of organization, and the time and space scales, i.e., the characteristic physical dimensions of the phenomenon under consideration (7). Therefore, the set of disciplines, and more generally the set of knowledge, we have to mobilize to deal with a specific IAH problem cannot be entirely defined *a priori* by a particular integrative framework. Rather it should be negotiated and incrementally agreed. Doing otherwise would freeze the definition of the problem and the set of perspectives to be involved in addressing it, limiting the set of possible responses and decisions. Therefore, the participatory process must be iterative and adaptive, focusing on clear and contextualized objectives. The participatory process enhances co-learning, evolves, and should strive to manage any conflict and power strategies (24, 26, 30). As much as possible, we must aim to create “safe forums for articulating and debating issues where facts are uncertain, values in dispute, stakes high and decisions urgent” (41–43).

From these considerations, a practical methodology for IAH should consider the three views of complexity, and should be iterative, adaptive, and participative (6).

## Systems Thinking as a Foundation

### Systems Theory and Modeling

Systems theory has a long history. The word theory is misleading here since it is more a paradigm than a theory one can falsify. Von Bertalanffy (44) in *General system theory* discussed the tendency of wholeness in sciences (holism), the necessity of knowledge integration, open and closed systems, feedback loops and control and regulation, interactions between system components and emergence (41, 45, 46). At its origin, systems theory is closely related to cybernetics (47, 48). It was J. W. Forrester who initiated the modern vision of the field of system dynamics. Forrester was a pioneer in applying systems engineering and computer simulations to analyze social systems and predict their behavior (49). Since, systems theory has spread and developed in different fields, such as business, management (50, 51) and ecology (52). We invite the reader to search the literature, e.g., Luhmann's 2012 book *Introduction to systems theory* (53), to grasp the depth of the field and explore its foundational developments. Systems theory provides a common language to deal with reality, necessary for collaborators to understand each other in interdisciplinary research. Von Bertalanffy used mathematics derived from the field of dynamical systems to describe systems and predict their behavior. The field of dynamical systems inherits insights from a long tradition in mathematics and physics in its methods and principles, which still widely support current modeling and simulation in biology, ecology, and epidemiology. Dynamical systems applications provide a fundamental theoretical framework IAH can benefit from, including, for example, perspectives derived from chaos theory or resilience theory (54). Theory of modeling and simulation (55) can be viewed as a foundation for more recent model variants developed in ecology, epidemiology and coupled social and ecological modeling, such as agent- or individual-based models (56–58). This has important implications for IAH development, which can benefit from the conceptual and methodological advances in these mathematical fields. Although modeling has been highlighted recently for its potential to deal with IAH complexity (59, 60), its use in the context of IAH research and intervention remains minimal. We emphasize here that modeling is a process of applying systems theory.

### Systems Thinking

**Figure 1:**
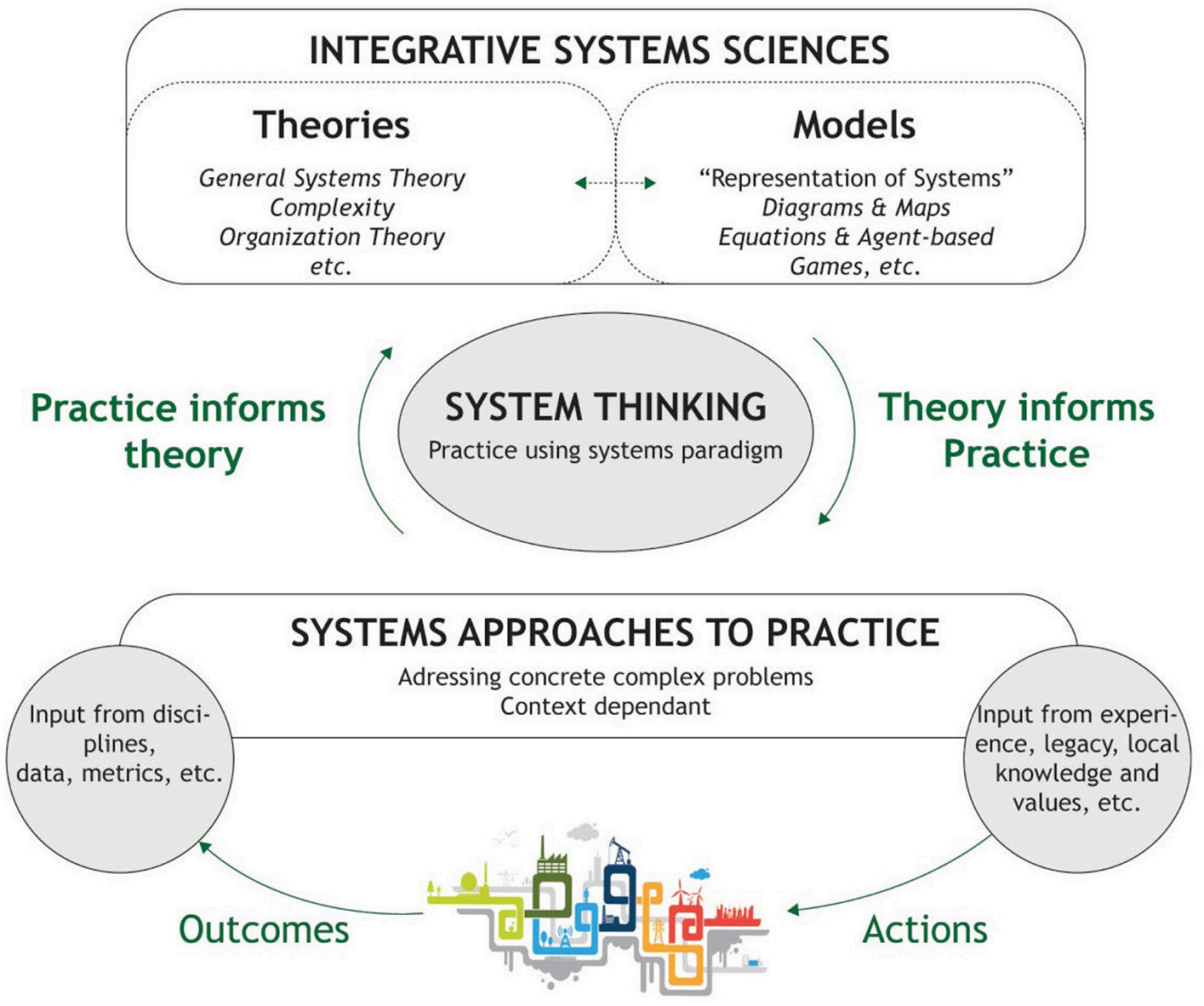
Systems thinking—Adapted from (65).

The term “systems thinking” has developed as an approach to real-world problem solving through the Operational Research field (41, 61–63). P. M. Senge developed the concept in the field of organizational theory and management. He identified the problems brought by fragmented knowledge and the lack of holistic learning in organizations (50). Ross and Wade (64) presented systems thinking as a set of skills used to improve the capability of identifying and understanding systems, predicting their behaviors, and planning change to produce desired effects. Systems thinking is a practice based on systems theory. It addresses concrete problems where the complexity of the system constrains understanding and explanation due to pre-conceptions and the limitations of cognitive processing. Figure 1 gives a synoptic view of systems thinking.

Ross and Wade (64) summarize the following sequence of tasks when applying systems thinking:

- Identify and understand the system structures considering different scales and levels.
  - Definition of the system boundaries, its closeness or openness, the scales considered in time and space, the organization level (cells, individuals, societies, ecosystems, etc.), the set of components and the connectivity between them.
- Identify and understand the system dynamics at different scales.
  - Description of the flows of matter, energy and information between the components, the synergies and the context-dependent changes in system structure.
- Reliably infer the impact of change to the system.

To these points we add elements of social context when applying systems thinking:

- Incorporate multiple perspectives and worldviews.
- Consider the environmental, cultural, religious, economic, and political contexts.
- Consider power relationships.

While these tasks are often used to describe modeling and simulation activities, they are increasingly used as a framework for complex participatory problem solving. Modeling activities are defined broadly and include a wide range of tools and methodologies (66). These include mind maps, charts, diagrams, or equations, while simulation ranges from computation using a computer to role playing games involving human actors. These existing tools support practitioners to apply modeling and simulation to implement systems thinking.

The knowledge we have about the context, the structure and the dynamics of a system constrains the decisions we make on its management, and consequently the potential for sustainable health development. When implementing IAH, knowledge is distributed and exchanged between different actors. Therefore, their participation and smooth communication is necessary if we want to integrate the contexts and the different perspectives into the iterative process of identifying problem and solutions (67). Modeling and simulation should therefore be participative and integrated research teams should also include social science expertise in engagement and facilitation for designing, managing and evaluating the participatory process (27, 68).

### Participatory Modeling and Co-learning

From the above, we argue that IAH should be iterative, adaptive and participatory to deal with complexity. Participatory modeling demonstrates how these requirements can be met in practice. It mobilizes the implicit and explicit knowledge of different actors to build a shared representation of reality (67, 69–72). Participatory modeling is a transdisciplinary process that facilitates knowledge sharing and the generation of new knowledge to support negotiation and planning. As such, it supports decision-making and adaptive management (73). The model is not presented as a singular definitive solution or final product but an intermediary pedagogical device used to foster dialogue (74). Numerous participatory modeling methods exist, all derived from the field of collaborative learning which appeared in the late 1960s (75). Co-learning is an approach that promotes multiple forums where diverse participants can work together on concrete problems or to create a product (76, 77). Discussions are not just about technical solutions, but also center on issues of ethics and power (31, 68). When coupled with participatory simulation (scenario analysis), this approach generates collective innovations.

Group Model Building (GMB), initiated in the field of system dynamics in the 1980s (67, 78, 79), was the first participatory modeling methodology studying stakeholder involvement and its effects on model production and decision-making, and fostering ideas into concrete actions. GMB was first applied in the business field, and environmental modelers adopted it in more diverse, cross-sectoral and ill-defined contexts characterizing environmental challenges (75). Methodologies, such as Community-Based System Dynamics modeling (CBSD) developed in 2009 by P. Hovmand (80), and Mediated Modeling (MM) founded in the 2000s by M. van den Belt (81) are closely related to the GMB approach. MM involves a series of workshops proceeding through stages of problem definition, conceptual model of the system (in which scientists may help to quantify flows and gather data), then participants “test” the model through scenarios. A strength within the mediated modeling approach developed by Thompson et al. (27) is that facilitators created a novel operating context for the modelers, using participatory techniques, such as historical and cultural timelines to elicit participant knowledge about changes in the complex system under study. Whereas CBSD focuses on advancing social innovation and capacity building, MM promotes more the creation of a shared understanding. These methodologies are mainly associated with systems thinking and facilitation, but fit very well with the development of system dynamics models.

Spatial group model building (28, 82) incorporates a spatial basis to participatory modeling, which can either be computer-assisted (28), or use manual methods, such as transparent map overlays (82) depending on the nature of the participants and availability of technology in the study context. Maps help participants visualize the context of the complex system, elicit more contextualized information (28), locate components of the complex system, and allow tracing of movements, such as supply chains (82).

Another participatory modeling approach that appeared in the 1990s, the Companion Modeling (ComMod) (72), shares many characteristics with the methodologies cited above. It uses multi-agents systems and role playing games to conduct participatory simulation sessions. ComMod systematically considers power relationships among stakeholders within a social context. It was characterized in a review paper by Seidl as a “genuine participatory approach” [(38), p. 575], ComMod is deeply adaptive; the model evolves as the problems change during the research and implementation period. Even the modeling tools can be different all along the process. Therefore, ComMod qualifies itself more as a research posture than a methodology attached to a particular set of tools.

On the basis of these different methodologies and others, a generic framework of participatory model development has been proposed (83). Binot et al. (59) and Duboz and Binot (84) contend that participatory modeling approaches are ideal to accompany adaptive management as well as to foster engagement and sharing of responsibilities. They should be used to implement IAH. However, Allen et al. (68) remind us that existing participatory initiatives in integrated science are better seen as islands of success, rather than evidence of a new sweeping paradigm.

In proposing participatory modeling as a potential pathway for improved systems approaches that can deal with the complexity of IAH we do not wish to under-state the potential shift in ethos and practice required. Participatory modeling itself needs to reside within an overall systems problem-solving framework. This calls for greater appreciation of the social processes of building collective systems knowledge, understanding choices and designing and monitoring interventions for change. This correspondingly calls for a wider remit for social process specialists in IAH initiatives (27) and, we would argue, an acknowledgment of the limitations of modeling approaches without this partnership.

## Conclusion

In this paper, we highlight systems thinking as a necessary foundation for IAH. We do so by discussing the origins and epistemology of systems thinking, revealing its strong mathematical roots and arguing that modeling informed by both dynamical system theory and social participatory innovations can help ground IAH theoretically and advance the tools necessary to deal with complexity in practice. Mathematical or computational models, for instance, within the first and second views of complexity (see Table 1) help identify and understand global system properties, such as feedback loops, controls, viability, resilience and emergence, elements of complexity that are usually ignored mostly because of the lack of tools to assess them. Combining mathematical models with innovative participatory approaches that encourage co-learning helps ensure that modeling is context-sensitive (i.e., culturally and socially relevant), iterative and adaptive. These approaches also enable a normative focus to ensure issues around ethics, equity, and power are included in the decision-making process. The idea of a negotiated complexity (85) for decision support systems similarly supports more inclusion of social sciences in embracing complexity. We argue that participatory modeling should be a key component of any IAH initiative, as it enables the practical operationalization of an otherwise elusive holistic effort.

To take a consistent and comprehensive approach, funding schemes supporting IAH would follow the same iterative and adaptive principles. It follows that funding agencies need to be included in the design and development of IAH research and adaptive management strategies. Furthermore, and despite the critical role played by participatory innovations that are inherently grounded in social sciences theory and practice, many key social science disciplines remain under-represented in most current IAH. Including social research expertise to manage appropriate participation, social engagement and facilitation is essential to address the ethical dimensions of systems, identify power relations, equity and gender issues, and therefore are keys to adapt the modeling process to the social and cultural context.

Accordingly, we argue that systems thinking and its attributes should be part of veterinary health and public health curricula (it is already well-accepted in ecology, the other dimension in One Health and EcoHealth). To achieve this, theory and methods taught in systems engineering and ecology can be adapted to the particular issues addressed by these domains. This instruction also needs to go further and provide students some expertise and familiarity in working comfortably in inter- and trans-disciplinary teams that also include system thinking specialists that can bring complementary skills in system dynamics modeling and in facilitation and problem structuring.

## Author Contributions

RD and PE are the main contributors. The manuscript is the result of a workshop where all the authors collectively decided the argument and content. All authors contributed ideas, passages and edits to the manuscript and actively reviewed and provided comments to the manuscript prior to submission.

### Conflict of Interest Statement

The authors declare that the research was conducted in the absence of any commercial or financial relationships that could be construed as a potential conflict of interest.

## Footnotes

^1^A “model” is a representation of reality (e.g., diagram, map, mathematics, game).
